# Revascularization After Traumatic Spinal Cord Injury

**DOI:** 10.3389/fphys.2021.631500

**Published:** 2021-04-30

**Authors:** Chun Yao, Xuemin Cao, Bin Yu

**Affiliations:** Key Laboratory of Neuroregeneration of Jiangsu and Ministry of Education, Co-innovation Center of Neuroregeneration, NMPA Key Laboratory for Research and Evaluation of Tissue Engineering Technology Products, Nantong University, Nantong, China

**Keywords:** spinal cord injury, blood vessel, nerve regeneration, therapeutic interventions, angiogenesis

## Abstract

Traumatic spinal cord injury (SCI) is a complex pathological process. The initial mechanical damage is followed by a progressive secondary injury cascade. The injury ruptures the local microvasculature and disturbs blood-spinal cord barriers, exacerbating inflammation and tissue damage. Although endogenous angiogenesis is triggered, the new vessels are insufficient and often fail to function normally. Numerous blood vessel interventions, such as proangiogenic factor administration, gene modulation, cell transplantation, biomaterial implantation, and physical stimulation, have been applied as SCI treatments. Here, we briefly describe alterations and effects of the vascular system on local microenvironments after SCI. Therapies targeted at revascularization for SCI are also summarized.

## Introduction

Traumatic injury to the spinal cord activates several complex pathological events, resulting in physical disability, psychological devastation, and social burdens (Hutson and Di Giovanni, 2019). Nerve tissues are damaged after spinal cord injury (SCI) with motor and sensory neuron dysfunction. Although the injured nerve tissues can undergo repair, the regeneration is limited and usually hypofunctional. Thus, there is an urgent need for interventions that effectively promote axon regeneration and functional recovery after SCI (Oudega, 2012). The acute SCI includes two phases, the primary mechanical destruction (immediate effect of trauma) and a later secondary injury (occurring over a time course from minutes to weeks; Silva et al., 2014). A cascade of progressive damages occurs in the lesion, such as vascular disruption, inflammation, demyelination, and apoptosis, leading to glial scar and cavity formation (Fleming et al., 2006; Garcia et al., 2016).

As mentioned, blood vessels that play a crucial role in nerve regeneration and functional recovery are ruptured. The mechanical forces destroy not only neural cells (neurons, astrocytes, and oligodendrocytes) but also blood vessels around the injury epicenter. In addition, the blood-spinal cord barrier (BSCB) in the surrounding tissues has increased permeability. This destruction induces ischemia and inflammation, which further exacerbates the overall tissue damage (Haggerty et al., 2018). Although new blood vessels form in the lesion, this angiogenesis is insufficient. Hence, understanding the vascular responses in the lesion microenvironment after SCI is of great importance. Interventions modulating vascular responses to promote sufficient functional vessel formation are needed for SCI therapy (Ng et al., 2011). To date, multiple treatments for blood vessel interventions in SCI have been developed, including proangiogenic factor administration, gene modulation, cell transplantation, biomaterial implantation, and physical stimulation. This review will provide an extensive overview of the vascular alterations after SCI and summarize current attempts to repair SCI using blood vessel interventions.

## Vascular Responses After Spinal Cord Injury

The vascular system consists of interconnected endothelial cell tubules and is a highly branched and hierarchically ordered network, similar to the nervous system (Serini and Bussolino, 2004). In normal conditions, the blood vessels transport ingredients (oxygen, nutrients, and hormones), remove metabolic waste and facilitate cell circulation, which provides a supportive microenvironment for the nervous system (Adams and Eichmann, 2010; Himmels et al., 2017). Anatomically, there is a higher density of capillary beds in the gray matter than in the white matter, and this may be to meet the greater demand for metabolic activity in neuronal cells within the gray matter (Haggerty et al., 2018).

Several phases of repair occur in the microenvironment of the lesion after SCI. These responses include regeneration-associated gene expression, axonal sprouting, oligodendrocyte remyelination, and endogenous angiogenesis. However, the effectiveness of this repair is limited (Hagg and Oudega, 2006). Glial scars, inflammation, growth-inhibitory molecules, and blood vessel disruption contribute to the hostile microenvironment at the injury sites, hindering axon regeneration and functional recovery (Silva et al., 2014; Tran et al., 2018). The initial mechanical force causes immediate local vascular damage and BSCB breakdown that increases vascular permeability. The ensuing ischemia and immune cell infiltration accelerate secondary anatomical damage and neurological deficits. As a response to decreased vessel density, endogenous angiogenesis and vascular remodeling take place around the lesion.

### Blood Vessel Rupture and Hemorrhage

Under healthy conditions, vessels and astrocytic processes associate closely to form the perivascular basement membrane (BM). With injury, mechanical trauma ruptures local microvasculature structure and disconnects blood vessels with astrocytes. The blood vessel wall then separates from the BM into an inner endothelial and an outer parenchymal part (Takigawa et al., 2010). There is a dramatic endothelial cell (EC) loss due to necrosis that occurs within the first 24 h after the insult. Then, in the following days, ECs undergo apoptosis induced chiefly by ischemia. Detachment from the extracellular matrix (ECM) surrounding the blood vessels also contributes to EC loss (Casella et al., 2002; Oudega, 2012). Blood vessel density around the lesion continues to decrease. Structurally altered BMs further exacerbate the expansion of inflammation during the subacute phase of SCI. Cellular debris from the disrupted vascular architecture is harmful to nearby neural cells, aggravating cell death. In the meantime, hemorrhaging occurs at lesion sites. The bleeding accelerates thrombin formation and increases extracellular glutamine levels, red blood cell lysis, and iron toxicity, which together exacerbate axonal damage (Losey et al., 2014). Hemorrhaging occurs chiefly within the gray matter and later extends radially into adjacent white matter (Tator and Koyanagi, 1997; Losey et al., 2014). Injection of bacterial collagenase, which minimizes mechanical injury, has shown that the induced hemorrhaging is associated with BSCB disruption, leukocyte recruitment, and axonal damage, leading to secondary injury and poor neurological outcomes after SCI (Losey et al., 2014).

### BSCB Disruption

The blood-spinal cord barrier (BSCB), a tight barrier between the blood and spinal cord tissues, assists in maintaining spinal cord homeostasis (Sharma, 2005; Yu et al., 2016a). The tight junction proteins, vascular basal lamina, astrocyte end-feet processes, and pericytes comprise the basal molecular structure of BSCB, similar to that of the blood-brain barrier (Huber et al., 2001). The initial mechanical injury force, combined with compression or vascular dilation-induced shear stress, disrupt the neurovascular unit and membrane structure (Oudega, 2012; Jin et al., 2021). Injury-induced pro-inflammatory cytokines (such as TNFα and IL-1β), vasoactive substances (such as reactive oxygen species, nitric oxide, and histamines), and matrix metalloproteinases (MMPs) elevate vascular permeability (Donnelly and Popovich, 2008). The BSCB disruption rapidly occurs in the first several hours. This abnormal permeability of BSCB is apparent during angiogenesis, which proceeds during a period of 3–7 days post-injury (Whetstone et al., 2003). The hyper-permeability of BSCB further damages local blood vessels. Immune cells, such as lymphocytes, neutrophils, and monocytes, infiltrate into the lesion site, leading to inflammatory responses. Calcium, excitatory amino acids, free radicals, and inflammatory mediators also pass into the injury site, contributing to secondary injury after SCI (Ng et al., 2011).

### Blood Supply/Ischemia

Traumatic injuries also affect spinal cord blood supply, which is closely correlated with the severity of damage (Martirosyan et al., 2011). The degenerated ECs sever local vascular networks, aggravating ischemia. The lack of adequate blood supply induces apoptosis and death of neural cells in the lesion epicenter (Figley et al., 2014; Losey et al., 2014). Hence, this reduction in blood flow leads to further tissue loss. Reduced blood pressure also decreases microvascular blood flow, accelerating organ dysfunction (Guha et al., 1989). Restoring blood supply around lesion sites is pivotal for SCI repair. Vascular smooth muscle cells (vSMCs) and pericytes around vessels coordinate to control blood flow into the central nervous system. It has been conclusively demonstrated by Li et al. that pericytes on capillaries caudal to the lesion abundantly express a vasoconstriction-relevant enzyme. Treatments that dilate vessels and improve blood flow facilitate functional recovery after SCI (Li et al., 2017).

### Endogenous Angiogenesis

Briefly, there are three main blood vessel formation mechanisms: vasculogenesis, splitting angiogenesis, and sprouting angiogenesis (Adams and Eichmann, 2010). Vasculogenesis is a process of generating vessels from endothelial precursor cells or angioblasts. This process mainly occurs in the early embryo at the vasculature development stage (Risau and Flamme, 1995; Flamme et al., 1997). In disease or injury repair situations, new vessels usually form from the existing vasculature, termed angiogenesis (Adams and Eichmann, 2010). Sprouting and splitting are two types of angiogenesis. In sprouting angiogenesis, the ECM of existing vessels is reorganized and paves ways for ECs. ECs then migrate, proliferate, generate tubules, and finally form new sprouts (Eilken and Adams, 2010). Splitting angiogenesis (or intussusception) is achieved by splitting from pre-existing vessels oppositely. New vessels then grow under the influence of growth factors, pericytes, and myofibroblasts (Gianni-Barrera et al., 2018).

Several molecules and signaling pathways participate in angiogenesis and endothelial regeneration (Tsuji-Tamura and Ogawa, 2018; Evans et al., 2021). Transcription factors, such as FoxM1 (Huang and Zhao, 2012), HIF-1α (Huang et al., 2019), Sox17 (Liu et al., 2019a) Atf3 (McDonald et al., 2018), and Foxo1 (Wilhelm et al., 2016) are involved in EC proliferation. Mef2 factors regulate sprouting angiogenesis (Sacilotto et al., 2016). Foxo1 and Foxo3 inhibit the migration and tube formation of HUVEC (Potente et al., 2005). These transcription factors integrate with multiple signaling pathways to modulate angiogenesis. VEGF signaling participates in angiogenic processes in both physiological and pathological conditions (Apte et al., 2019). PI3K-Akt signaling activation enhances HIF-1α production, increases VEGF expression, and promotes angiogenesis (Jiang et al., 2000, 2001). Inhibition of the PI3K-Akt and mTOR signaling pathways can activate Foxo1 and induce EC elongation (Tsuji-Tamura and Ogawa, 2016). Additionally, Notch signaling is also important during angiogenesis. Dll4, which is expressed at tip cells, can bind to and activate Notch signaling in neighboring stalk cells to modulate sprouting and branching (Hellstrom et al., 2007; Eilken and Adams, 2010).

Angiogenesis is the primary form of vascular formation in the lesion microenvironment after SCI. Triggered by hypoxia and proangiogenic growth factors, ECs undergo sprouting, proliferation, and finally remodeling (Carmeliet, 2000, 2003). It is possible that angiogenesis serves as an early scaffold for axonal regeneration across the injury site, facilitating tissue remodeling and survival (Casella et al., 2002). Blood vessel density transiently increases within 2 weeks due to endogenous angiogenesis. However, endogenous angiogenesis is insufficient to support local metabolism, accelerating hypoxic ischemia and cell death at lesion sites. Additionally, newly formed blood vessels are usually leaky with impaired BSCB (Fassbender et al., 2011). New vessels fail to associate with other cells (neurons, astrocytes, or pericytes) and do not organize into a functional vasculature (Loy et al., 2002; Ng et al., 2011). Due to their geometry and plasticity, the new vessels do not guide neighboring sprout outgrowth (Losey et al., 2014). The malfunctioning vasculature impedes self-repair at the injury site and prevents functional recovery after SCI.

## Neuroprotective Effect of Blood Vessels

There is a close relationship between revascularization and improved functional outcomes after SCI. First, a well-vascularized lesion provides a permissive microenvironment for local tissue survival and nerve regeneration. It has been demonstrated that improved capillary blood flow (Li et al., 2017), angiogenesis (Hu et al., 2015), and BSCB integrity (Lu et al., 2019) can facilitate functional recovery after spinal cord injury. Blood vessels potentially serve as a scaffold to guide axonal sprouting after injury. Emerging evidence has shown that there are similarly attractive and repulsive cues in vascular and axonal guidance, such as Ephrins, Semaphorins, Slits, Nogo, and VEGF (Serini and Bussolino, 2004). Blood vessels and nerves interact physically and can affect each other. VEGF-A, released from neurons and glial cells, promotes vessel growth (Ferrara, 2005). Neurotrophins, such as NGF and NT-3, control the sympathetic innervation of blood vessels. Moreover, vascular cell types, such as vSMCs, secret endothelins, providing guidance cues for axons (Ieda et al., 2004). Other research has shown that ECs express a repulsive axon guidance cue termed Semaphorin 3A, which inhibits axonal growth to blood vessels (Damon, 2006). Besides neurons and axons, blood vessels also interact with other cells, including astrocytes, microglia, and oligodendrocytes. After SCI, EphA4, for example, is upregulated in astrocytes that are tightly associated with blood vessels. Silencing EphA4 decreases the tight association between astrocytes and blood vessels, impairing BSCB (Goldshmit et al., 2006). The vascular endothelium also provides a physical substrate for oligodendrocyte precursor migration (Tsai et al., 2016). Recent research indicates that microglia play a critical role in maintaining vascular integrity *via* fibrinogen-Mac-1 interaction (Halder and Milner, 2019).

In general, revascularization is essential for nerve repair. Therapeutic interventions for SCI can focus on the lesion vasculature. Approaches to promote angiogenesis, restore blood supply, and regain a non-leaky state vascular system as early as possible will attenuate secondary damage, limit nerve tissue loss, promote axon regeneration, and improve functional recovery after SCI.

## Therapeutic Interventions

**Figure 1:**
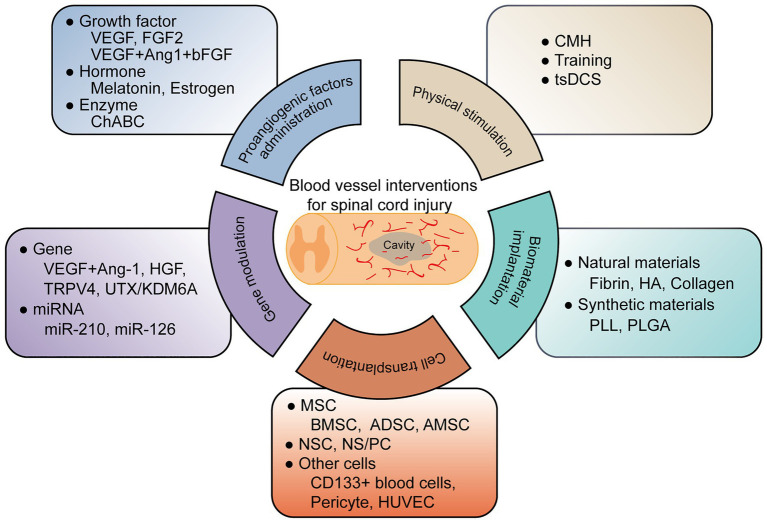
A graphic summary of current blood vessel interventions for spinal cord injury.

To date, researchers have conducted several therapeutic interventions based on revascularization for SCI. These treatments include proangiogenic factor administration, gene therapy, cell transplantation, biomaterial implantation, and physical stimulation, illustrated in Figure 1 and Table 1.

**Table 1:** Lists of current blood vessel interventions for spinal cord injury.

Proangiogenic factors administration	
Growth factor	VEGF(Widenfalk et al., 2003), VEGF+Ang1+bFGF (Yu et al., 2016b), FGF2 (Kang et al., 2010, 2013)
Hormone	Melatonin (Wu et al., 2014; Jing et al., 2017), Estrogen(Samantaray et al., 2016; Ni et al., 2018)
Enzyme	ChABC (Milbreta et al., 2014)
Others	FFA (Yao et al., 2018b), MMP-8I (Kumar et al., 2018), G-CSF (Kawabe et al., 2011)
Gene modulation	
Gene	VEGF+Ang-1(Herrera et al., 2010), HGF(Kitamura et al., 2007), TRPV4 (Kumar et al., 2020), UTX/KDM6A (Ni et al., 2019)
miRNA	miR-210 (Ujigo et al., 2014), miR-126 (Hu et al., 2015)
Cell transplantation	
MSC	BMSC (Matsushita et al., 2015; Watanabe et al., 2015; Ropper et al., 2017), ADSC (Zhou et al., 2013), umbilical cord (Badner et al., 2016), AMSC (Zhou et al., 2016)
NSC, NS/PC	NSC(Kim et al., 2009; Lien et al., 2019), NS/PC(Kumagai et al., 2009; Nori et al., 2011), NPC+EC (Rauch et al., 2009)
Other cells	CD133^+^ peripheral blood cells (Fujioka et al., 2012; Sasaki et al., 2009), Pericyte (Badner et al., 2016), HUVEC (Zhong et al., 2020)
Biomaterial implantation	
Natural materials	Fibrin (Yao et al., 2018a; Loureiro et al., 2019), HA (Wei et al., 2010), Collagen (Wang et al., 2018)
Synthetic materials	PLL (Patist et al., 2004), PLGA (Yu et al., 2016b; Ropper et al., 2017)
Physical stimulation	
	CMH (Halder et al., 2018), Training (Kissane et al., 2019; Ying et al., 2020, 2021), tsDCS (Samaddar et al., 2017)

### Proangiogenic Factor Administration

A widely accepted vascular intervention for SCI is that of regulating angiogenic factors (Klagsbrun and Moses, 1999; Graumann et al., 2011). Vascular endothelial growth factor (VEGF), is one of the best characterized angiogenic factors, which modulate blood vessel formation, promote the proliferation and migration of ECs (Ferrara et al., 2003). VEGF treatment alone (Widenfalk et al., 2003) or in combination (Yu et al., 2016b) increases angiogenesis and axon regrowth. Delivery of angiogenic factor fibroblast growth factor-2 (FGF2) into the lesion site elevates blood vessel density, increases blood flow rates, and decreases the permeability of BSCB. However, the improvement in axonal and functional recovery is not apparent (Kang et al., 2010, 2013).

Some hormones (melatonin, estrogen; Wu et al., 2014; Samantaray et al., 2016; Jing et al., 2017; Ni et al., 2018), enzymes, and chemical drugs also have a proangiogenic effect and are applied in SCI therapy. Chondroitinase ABC (ChABC) is widely used in SCI treatments to degrade extracellular chondroitin sulfate proteoglycans (CSPG), a primary obstacle for axon regeneration. Researchers have shown that ChABC also stimulates axonal remodeling by promoting revascularization (Milbreta et al., 2014). Cleavage of CSPG by ChABC affected the detachment and separation of blood vessel BM, enhancing neoangiogenesis and blood vessel maturation (Milbreta et al., 2014). MMPs create a hostile environment for SCI recovery. Treatments using flufenamic acid (FFA) or the specific MMP-8 inhibitor (MMP-8I) significantly attenuate MMP-mediated BSCB disruption (Kumar et al., 2018; Yao et al., 2018b). Colony-stimulating factor G-CSF (Kawabe et al., 2011) promotes local angiogenesis by increasing angiogenic cytokine expression.

### Gene Therapy

Proangiogenic factors overexpressed by viral vectors have been shown to improve angiogenesis and enhance BSCB integrity after SCI (Kitamura et al., 2007; Herrera et al., 2010). Additionally, genes dysregulated after SCI may also be potential targets for blood vessel innervations. For example, transient receptor potential channel protein TRPV4 increases during the acute phase of SCI. TRPV4 KO mice show reduced EC damage, increased tight junction proteins, and attenuated inflammation, leading to improved neuroprotection and functional recovery (Kumar et al., 2020). Knockdown of UTX, a histone H3K27 demethylase that is upregulated in ECs after SCI, promotes EC migration and tube formation. Functional recovery is also enhanced as evaluated by BMS score, electrophysiology, tactile and temperature sensation (Ni et al., 2019). Mechanism analysis shows that UTX decreases promoter methylation of miR-24, which targets genes involved in angiogenesis (Ni et al., 2019).

MicroRNAs (miRNAs), such as miR-210 and miR-126, also participate in angiogenesis (Fish et al., 2008; Kir et al., 2018). Administration of miR-210 increases blood vessels at day 3 after SCI, probably by inhibiting the expression of PTP1B and EFNA3, two antiangiogenic factors in proper vascular growth (Ujigo et al., 2014). Agomir-126 treatment after SCI results in enhanced vascularity, reduced inflammation, and improved locomotor function. Mechanism analysis suggests that this angiogenic effect of miR-126 is mediated by repressing SPRED1 and PIK3R2 (Fish et al., 2008; Hu et al., 2015).

### Cell Transplantation

Mesenchymal stem cells (MSCs) are capable of self-renewal and differentiation. It is an attractive cell source for cell-based therapeutic strategies (Assinck et al., 2017). Transplantation of MSCs derived from bone marrow (BMSCs; Matsushita et al., 2015; Watanabe et al., 2015; Ropper et al., 2017), adipose tissue (ADSCs; Zhou et al., 2013), umbilical cord (Badner et al., 2016), or amnion (AMSCs; Zhou et al., 2016) have shown pleiotropic positive effects on the lesion microenvironment after SCI, including increased angiogenesis and restored BSCB integrity. This probably is due to the angiogenic factor secretion by MSCs (Baraniak and McDevitt, 2010). However, there are some limitations on MSC treatments, such as insufficient reach to the lesion core and potential tumor formation (Lu et al., 2019). Extracellular vesicles or exosomes derived from MSC contain paracrine-secreted angiogenic factors. They can exert the role of MSCs in neovascularization, improving SCI recovery (Kim et al., 2018; Lu et al., 2019; Huang et al., 2020).

Neural stem cells (NSCs) or neural stem/progenitor cells (NS/PCs) can differentiate into three major neuroglial lineages (neurons, astrocytes, and oligodendrocytes). Their implantation has an angiogenic effect induced by elevated VEGF expression in the injured spinal cord tissue (Kim et al., 2009; Kumagai et al., 2009; Nori et al., 2011). The astrocytic components of NSC grafts can also migrate and join endogenous astrocytes, integrating into the host BSCB (Lien et al., 2019). Co-implantation of NPCs and ECs after SCI increases functional vessel density and promotes BSCB re-establishment (Rauch et al., 2009).

Other cells, such as CD133^+^ peripheral blood cells (Sasaki et al., 2009; Fujioka et al., 2012), pericytes (Badner et al., 2016), and HUVECs (Zhong et al., 2020), also have proangiogenic effects on SCI repair. However, given the pleiotropic effects of cell transplantation on the spinal cord, improved vascular function may not be a significant cause of the observed functional recovery.

### Biomaterial Implantation

Natural or synthetic biomaterials support revascularization by promoting and guiding blood vessel formation (Haggerty et al., 2018; Liu et al., 2019b). Fibrin, hyaluronic acid (HA), and collagen are natural biomaterials with intrinsic angiogenic properties. They can combine angiogenic factors to enhance vascularization after SCI (Wei et al., 2010; Wang et al., 2018; Yao et al., 2018a; Loureiro et al., 2019). Synthetic biomaterials, such as poly (L-lactic acid; PLL; Patist et al., 2004) and poly (lactic-co-glycolic) acid (PLGA; Yu et al., 2016b; Ropper et al., 2017), additionally, can provide for sustained release of pro-angiogenic factors or delivery of implanted cells to the lesion site after SCI. These applications of biomaterials in angiogenesis for SCI have recently been comprehensively reviewed (Haggerty et al., 2018).

### Physical Stimulation

Treatments that induce hypoxia, a pro-angiogenic condition, also trigger the vascular remodeling response and promote vessel density after injury (Halder et al., 2018). Chronic mild hypoxia (CMH; 8% O_2_ for up to 7 days) promotes EC proliferation, vascularization, and BSCB integrity through α5β1 integrin. Locomotor training combined with epidural stimulation, moreover, increases capillary distribution across the muscle after SCI (Kissane et al., 2019). Recently, researchers have found that water treadmill training (TT) reduces BSCB permeability after SCI by inhibiting MMP-2/9 expression (Ying et al., 2020). TT also triggers the BDNF/TrkB-CREB signaling pathway to ameliorate BSCB disruption following SCI (Ying et al., 2021). Besides, trans-spinal direct current stimulation (tsDCS) promotes blood flow to modulate neural cell migration and proliferation after SCI (Samaddar et al., 2017).

## Conclusion and Perspective

Blood vessel vascularization and remodeling after SCI are critical for SCI repair and functional recovery. Researchers have applied numerous blood vessel interventions for SCI. However, the therapeutic effect is limited and the regulatory mechanisms are unclear. Several issues need to be addressed in further studies. First, there is the discovery of more potential genes or molecules. The vascular system and the central nervous system share many guidance molecules, such as Ephrins, Semaphorins, Slits, and Netrins. Manipulations of these genes will potentially promote vessel and axonal regeneration in SCI repair processes (Adams and Eichmann, 2010). Blood vessel interventions used in other diseases, such as stroke (Wang et al., 2004), Alzheimer’s Disease (Zlokovic, 2005), or peripheral nerve injury (Sekiguchi et al., 2012), provide another choice for SCI therapy. Second, there is the determination of interactions between nervous, immune, and vascular systems. As detected by two-photon microscopy, blood vessels transiently increase and interact with neurons within 2 weeks after SCI. However, the guidance of new vessels on adjoining sprouts is inadequate (Dray et al., 2009). Inflammation responses also regulate angiogenesis during tissue regeneration (Donnelly and Popovich, 2008). Macrophages can secrete factors to promote tip-endothelial cell fusion, remodel the basal membrane, and attract pericytes or vSMCs for revascularization (Haggerty et al., 2018). Finally, there is the identification of combined strategies for revascularization. As summarized, we can conduct blood vessel interventions for SCI by proangiogenic factor administration, gene modulation, cell transplantation, biomaterial implantation, and physical stimulation. Multiple strategy integration might strengthen the proangiogenic effects, restore BSCB, and overcome delivery limitations.

## Author Contributions

CY and XC wrote the manuscript. CY and BY reviewed the manuscript. All authors contributed to the article and approved the submitted version.

### Conflict of Interest

The authors declare that the research was conducted in the absence of any commercial or financial relationships that could be construed as a potential conflict of interest.
